# Hospital and Institutional News

**Published:** 1920-07-10

**Authors:** 


### HOSPITAL AND INSTITUTIONAL NEWS.

### SIR CLIFFORD ALLBUTT'S PORTRAIT.

At the conclusion of the address outlined above the company adjourned to King's College, where a reception was held and the portrait of the President presented to him by Sir Norman Moore, Bart., President of the Royal College of Physicians, London. The excellent portrait itself, by Sir William Orpen, R.A., was on view in King's College Hall, and, as was explained, it was not the gift of a particular Association, however distinguished, or a particular college or a particular set of men, for it carried with it the appreciation of the whole medical profession. It was aptly said that if Sir Clifford Allbutt had been living in the time of Rembrandt, Rembrandt would have been employed to paint him, but as it was, the best painter of masculine portraits had been asked to fulfil the task, and those who have been fortunate enough to see the picture will unanimously agree upon the artistic excellence of the work. Sir Clifford Allbutt, in receiving this gift, expressed his appreciation in characteristic and well-chosen words, and it must be with pleasure that the many men who subscribed to the presentation fund heard that the President felt, apart from the great honour they had just accorded to him in the Senate House, that the medical profession of the country had now done still more to set him upon what he might call the summit of a very long and happy life.

### APPRECIATION AND DISCRETION.

Many of the decisions reached and the comments made at Cambridge are reviewed elsewhere in our present issue (see pp. 381, 382), but the most noticeable feature of the meeting has probably been the absence of unreasoning criticism, which might have been expected to arise from some quarters upon the variety of new medical proposals now before the country. The Interim Reports of the Consultative Councils were under consideration, and the chief requirement of the times is that the great activities which these bodies have displayed should be appreciated. Their detailed work has yet to be done, and the degree of reserve with which all discussions were carried on was in every way calculated to assist them in forming satisfactory and matured conclusions. It was, however, very definitely decided that the contents of remedies administered by the medical profession must be disclosed to the profession, and the representatives were emphatic in their uncompromising refusal to entertain the idea of a whole-time State medical service.

### MR. MORRIS DISCUSSES THE POSITION OF HONORARIES.

Mr. Morris, of the London Hospital, speaking at the British Medical Association meeting, said that he firmly believed that the day of staffing voluntary hospitals by an honorary medical and surgical staff had gone by, at least in respect of the teaching hospitals. Clearly, in the old days, when the consultant treated his patients from his own personal experience, things were different. But now he does not fight disease alone. The fight is carried on by officers of all arms—many new arms. The complexity of the study of disease, the multiplicity of departments in combating it, demands more time to be spent in clinical work at the bedside than can be demanded of an honorary staff. Is the walk of a couple of hours twice a week through forty or fifty beds the ideal way of fighting the diseases represented? Is any disease to be studied and destroyed in that way? Can the bacteriologist, the bio-chemist, the clinical pathologist carry out research of any value by such means?

### THE GAMBLE OF THE CONSULTANT.

Mr. Morris drew a picture which showed that from the personal point of view of the consultant, possible success in his career is, under the present system, very much of a gamble. As a young man he qualifies. He holds the usual house appointment—clinical assistant to out-patients, house-physician, house-surgeon, resident accoucheur, and so on. Then he begins to get anxious as to his future. He looks up the probable dates of retirement of the honorary staff—a staff which is not likely to be enlarged, however much the work increases. He sees a chance, if he has enough capital to hang on. He becomes registrar at a starvation wage—one lower than a porter or stoker, but he is really paid by a promissory note, one drawn not in a financial sense upon the resources of the hospital as it happens. He finishes his registrarship and still hangs on as a demonstrator or what not until at long last he gets his appointment, which he will hold for twenty or thirty years, in fact for the rest of his working life. Mr. Morris thinks this is not a sound system, although it has produced such splendid men, because an appointment for life on a staff rigidly restricted in numbers ensures only to a few, instead of to the many, the tremendous advantage of being attached in this way to a great hospital.

### DINNER TO DR. C. H. MAYO.

The Chairman (Lord Reading) and the Committee of the projected American Hospital in London entertained to dinner on July 7 at Claridge's Hotel a large gathering of leading British medical men, and, as guest of honour, Dr. C. H. Mayo, of Rochester, Minnesota. In responding to the toast of his health, Dr. Mayo predicted that the next war will be, to a far greater extent than the last one, a war of scientific chemistry and applied pathology. He expects that the microbe will be pressed into service as a far deadlier weapon than any shell can be, and that the immolation of whole populations by disease purposely disseminated by their enemies is one of the contingencies which civilisation has to face. Mr. Balfour proposed the toast of the American Hospital in felicitous terms; it is not yet decided, apparently, where the hospital is to be located, and indeed it is hardly possible to draw up any plans until the amount of money available is approximately known. There are limitations of the wealth and generosity even of American cousins, and coats will have to be cut according to the cloth.

### THE KING'S FUND TO THE RESCUE.

The best financial news of the hospital world this week is of the emergency grant of £250,000 by King Edward's Hospital Fund for London to relieve the urgent difficulties of the Metropolitan hospitals. We understand that although this sum has been set aside for the purpose announced, not all the hospitals have yet actually received their grants. But the cheques received last Tuesday morning, whether representing the whole or part of the grant, must have been welcomed as a practical means to help solve the problem that confronts the committees. The public must not, however, be allowed to fall into the error that the money troubles of the hospitals are over or even substantially overcome. We understand, indeed, that it is the King's Fund's intention to help the hospitals to help themselves and to give them the courage to be up and doing. To rely exclusively upon the Central Funds to pay deficits over and over again is foolish policy. The Funds have not sufficient money to do it, and unless, what is not impossible, there should be a consolidation of effort in appeal, could not be a sole source of revenue. But what has been done by the Fund, after the drastic step of encroaching upon capital has been taken, is a bold and, we think, a wise step. It is now for the hospitals to show a better face to the present adverse outlook and bring home to the public its obvious duty in seconding the action of the King's Fund.

### A QUARTER OF A MILLION.

We understand the financial position of the hospitals is to be again reviewed by the King's Fund in October next, when the figures of the first three quarters of the year will be ready for comparison with those of 1919. Hospitals are asked to look both to the income and expenditure sides of the account when considering what is to be done. Amongst possible sources of income are suggested the organisation of contributions from employers or from employed, and from patients who can afford to pay. Hospitals are advised to review any necessary and practicable steps to keep expenditure within the available or prospective income, by postponing bed extensions and by exercising more stringent control over cost of working. Committees are asked to communicate to the Fund in due course particulars of any action which they may decide to take. The King's Fund, late in the day though some may think, has evidently taken the situation fully in hand, and they are to be congratulated upon the important and effective action that has been decided upon. Amongst the most important grants to hospitals the following may be quoted from a list including over 100 institutions: The London £35,000, King's College £17,500, Great Northern Central and St. Thomas's £12,000 each, the Metropolitan £11,000, Middlesex Hospital and University College £10,000 each, Westminster and the West London £9,500 each, St. George's £8,500, the National Hospital for the Paralysed and Epileptic and Hospital for Sick Children £8,000 each, Guy's and Prince of Wales's £7,000 each, Queen's Hospital for Children £6,500, and Queen Mary's and the Royal Free £6,000 each, the remaining grants ranging from £25 to £4,000.

### SURGEON-GENERAL W. G. GORGAS.

We regret to announce the death of Surgeon-General Gorgas, of the United States Army, which took place on July 4 at the Queen Alexandra Nursing Home, Millbank. General Gorgas had for many years devoted himself to the great combat with yellow fever in the New World, and the elimination of this disease in Cuba, Panama, and Havana stands as one of his many valuable achievements. A permanent director of the International Health Board of the Rockefeller Foundation, he was on his way out to the West African coast to inaugurate further yellow fever investigations when his fatal illness overtook him. His death robs the world of a practical benefactor to mankind.

### THE LAMBETH MEDICAL DEGREE.

The Archbishop of Canterbury has felt compelled to refuse the prayer of the petition signed by over 300 past and present members of Parliament that he should confer a Lambeth medical degree upon Mr. H. A. Barker "in view of his unique and distinguished services to suffering humanity and the cause of science through a long period of opposition, contumely, and persecution."

### THE MILLER HOSPITAL EXTENSION.

The extension scheme for the Miller General Hospital, Greenwich, which has been prepared by Sir Reginald Blomfield, R.A., and Mr. W. A. Pite, will be divided into two parts. On the site already obtained three ward-blocks, an out-patient department and the main part of the nurses' home can be built. Only the essential part of the scheme will be undertaken at present. So far £30,000 has been raised, but the price of building and labour continues to advance. In connection with the efforts made to increase the income, one dismal fact deserves mention. Not long ago an appeal was made to 200 churches in Greenwich, Deptford, Lewisham and Woolwich. The number of churches which held a collection was seven. On the other hand, the tradesmen and the schools have done comparatively well, and it is hoped that the building fund may receive some support from the War Memorial Schemes of Greenwich and Deptford. The Congregational Church in Greenwich Road, which, as The Hospital mentioned at the time, was purchased, will be eventually removed. While, we understand, the new administrative block will ultimately occupy the site, Mrs. E. S. Stidolph, noticing the discomfort of waiting out-patients, has given £1,000 towards the adaptation of the church to a shelter for them. It is extraordinary the want of consideration shown in the past to out-patients everywhere, who had to pay dearly for their treatment in hours of delay, and often enough in want of food or even protection from the weather.

### ANY OFFERS FOR A FULLY EQUIPPED HOSPITAL?

The Third London General Hospital at Wandsworth, which did such magnificent work during the war, is about to be closed by the War Office, and by the end of July the staff will be scattered and the equipment returned to store. The Ministry of Pensions has had the offer of this hospital and has not accepted it, although there are 15,000 discharged disabled men in the voluntary hospitals at the present time. A correspondent of *The Times* asks if it is economical to scrap a hospital such as this when it is absolutely certain more accommodation must be provided in the very near future. In the neighbourhood in which the Third London is situated there is nothing like enough hospital provision for the poor population, and this hospital requires hardly any adaptation for civilian patients and, temporary building though it is, would with reasonable care last for twenty years. The Third London is one of the few military hospitals in the London district housed in temporary buildings, the others were in schools and other institutions which have been returned to their former uses.

### CLOSING HOSPITAL WARDS.

Formerly the threat to close hospital wards produced as a rule little emotion. "Wolf" had been cried too often. But now there is an uncomfortable feeling that the act may actually follow the threat. The National Hospital for the Paralysed and Epileptic in Queen Square has discharged about twenty-five per cent. of the patients, and the remainder as their condition allows will shortly also be sent to their homes. The large out-patient department is still open, but it is little use seeing out-patients in a hospital of this kind unless there is a chance of obtaining beds for those that need them. The London Fever Hospital is another institution which is at its last financial gasp and had no money at the end of June to pay either wages or bills for the month. It is announced that the whole institution will be closed (there is no out-patient department involved) if help is not at once forthcoming. The Kensington and Fulham General Hospital is in a tragic position, and the rich neighbourhood it serves is unable to supply the sum necessary—less than £5,000—to keep the doors open.

### HOSPITALS AND THE FREE CHURCH COUNCIL.

WITH commendable public spirit the Brighton and Hove Free Church Council has held an ambitious bazaar at Hove Town Hall to help the Royal Sussex County Hospital reduce its overdraft. The bazaar, which lasted for three days and included a flag day, raised about £1,200. This sum included no large donations, but was composed of small contributions; and the programme which was issued for the function showed much care. Edited by Mr. William J. Cole, it took the form of a handbook, which began with a message from the Rev. F. B. Meyer, President of the Free Church Council, and included an excellent statement by the Rev. Frank J. Gould. He began well by reminding his readers that Hospital Sunday was ecclesiastical in origin, and gave an interesting sketch of the part played by the Free Churches in the mission field and in the social reforms of the nineteenth century. Then follows a history not only of the hospital, but of the interest in it shown by the inhabitants, which remind us of all the changes which Brighton has experienced since 1824, when the Earl of Egremont and Mr. Thomas Kemp (commemorated in the district known as Kemp Town) came to the rescue in the days of the coaching period. The programme of stalls and entertainments concluded with a sketch of most of the Free Churches in the town. There seems to have been sufficient energy to justify the hope that this function might be held yearly. We hope that Mr. L. Lancaster-Gaye, secretary of the Royal Sussex County Hospital, may agree, and that the Free Churches will unite to raise £1,000 a year for the hospital.

### REOPENING OF THE WEIR HOSPITAL, BALHAM.

THE Weir Hospital at Balham has at last been opened for civilian patients. It sprang from a bequest left by the late Mr. Benjamin Weir, a well-known local resident, who died in 1902. He left the bulk of his property to trustees to build and to maintain a cottage hospital for the inhabitants of the parish of Streatham and neighbourhood, but it was not until 1911 that the Charity Commissioners sanctioned a scheme authorising the trustees to build a hospital on the site of Mr. Weir's residence. This was duly carried out, and provided thirty beds. The building was just finished when the war broke out, and, as there was a difficulty in obtaining the requisite staff, the trustees decided to lend the hospital to the Kensington Division of the Red Cross Society. Before the end of the war 4,500 wounded soldiers passed through the hospital. Now that once again it has reverted to its original use there is much to be done ere it reaches perfection. For instance, although there is an *x*-ray room there is no apparatus, and only a promise of £150 towards the cost of £750 has as yet been received.

### AN ARMY RECORD.

MR. WINSTON CHURCHILL, on behalf of the Army Council, forwarded a certificate of the use to which the hospital was put during the war. This reads as follows: "Dieu et mon droit. During the Great War of 1914-9 this building was established and maintained as a hospital for British sick and wounded. The Army Council, in the name of the nation, thank those who have rendered to it this valuable and patriotic assistance in the hour of its emergency, and they desire also to express their deep appreciation of the whole-hearted attention which the staff of this hospital gave to the patients who were under their care. The War has once again called upon the devotion and self-sacrifice of British men and women, and the nation will remember with pride and gratitude their willing and inestimable service."

### THE BIRMINGHAM HOSPITAL SATURDAY.

MUCH pleasure has been felt in Birmingham at the fact that the Hospital Saturday Fund once again surpasses previous totals, and was expected (we note the prophecy) to reach between fifty and sixty thousand pounds. In 1919 £43,000 was raised. It is a fact that the number of contributors has increased, that the average annual ratio of progression has been surpassed this year; and that the increase is the result of the decision, made eighteen months ago, to raise the weekly contribution from one penny to twopence. Satisfactory as the gross increase is, the facts remain (*a*) that 1d. is now worth ½d., or perhaps somewhat less; (*b*) that the incomes of the working classes in organised industry have, as a rule, more than doubled. Consequently the real test of adequacy for the Saturday Fund's contribution in 1920 is to take double the total raised in 1918 as the minimum to be expected. In fact, we should expect more, because not only has the rate of contribution been doubled in the meantime, not only are the contributors better off, but there are *more* contributors than there were two years ago.

### THIS WEEK'S DRUG MARKET.

THE depression in the drug trade continues, and prices still show a strong tendency to sag. The downward movement indeed seems to be becoming more pronounced as buyers continue to hold off, and sellers in some cases are getting tired of waiting for business. Among the articles whose prices have moved downwards are English refined camphor, olive oil, cloves, castor oil, phenazone, sugar of milk, star aniseed oil, citric acid, cream of tartar, lemon oil, paraldehyde, and guaiacol carbonate. This by no means exhausts the list, however, and many other articles are in a weak market position. The price of methylated spirit has been advanced about 25 per cent. At the recent public sale of drugs in Mincing Lane the offerings were very heavy, but the demand was exceedingly quiet, and in the comparatively few cases where sales were effected prices were generally lower.

